# The two-component system histidine kinase LiaS contributes to the stress resistance and virulence of zoonotic *Listeria monocytogenes*

**DOI:** 10.1080/21505594.2026.2707724

**Published:** 2026-07-21

**Authors:** Yongshu Wu, Jiali Xu, Yifan Wang, Laiyin Hu, Mengyuan Gao, Shuyun Li, Mianmian Chen, Binjie Zhu, Lingli Jiang, Houhui Song, Changyong Cheng

**Affiliations:** a Key Laboratory of Applied Biotechnology on Animal Science & Veterinary Medicine of Zhejiang Province, Zhejiang Engineering Research Center for Veterinary Diagnostics & Advanced Technology, Zhejiang International Science and Technology Cooperation Base for Veterinary Medicine and Health Management, Belt and Road International Joint Laboratory for One Health and Food Safety, China‑Australia Joint Laboratory for Animal Health Big Data Analytics, College of Veterinary Medicine, Zhejiang A&F University, Zhejiang, Hangzhou, China; b Ningbo Key Laboratory of Skin Science, Ningbo College of Health Sciences, Ningbo, Zhejiang, China

**Keywords:** *Listeria monocytogenes*, two-component system, histidine kinase, stress resistance, bacterial pathogenesis

## Abstract

LiaS is a histidine kinase receptor in two-component systems that senses environmental stress signals. This study investigated the roles of LiaS in *Listeria monocytogenes* stress resistance and virulence. Phenotypic assays showed that deletion of *liaS* (Δ*liaS*) resulted in significantly impaired growth under acidic, alkaline, and osmotic stresses, as well as reduced survival under strong acidic conditions. These defects were partially restored in the complemented strain (CΔ*liaS*). The growth defects were also observed in the Δ*liaS* under Cu^2+^ or H_2_O_2_ exposure. Furthermore, the Δ*liaS* strain exhibited impaired invasion and intracellular migration in host cells, along with attenuated colonization in the liver and spleen at 24 h post-infection. Bacterial loads in the spleen of infected mice remained lower at 48 h. The mortality was also delayed in Δ*liaS*‑infected mice. Transcriptional analysis revealed that compared to neutral conditions, osmotic stress-related genes (except *opucB* and *gbuC)* were significantly upregulated in the Δ*liaS* strain under acidic conditions, indicating that LiaS modulates acid tolerance through transcriptional regulation. Collectively, LiaS is essential for the adaptation of *Listeria monocytogenes* to acidic, osmotic, and oxidative stresses. Its absence attenuates host cell invasion, intracellular motility, and organ-specific colonization, highlighting its dual role in environmental resilience and pathogenicity. These findings provide a theoretical basis for understanding host–pathogen interactions and offer new strategies against antimicrobial-resistant pathogens.

## Introduction

*Listeria monocytogenes* (*L. monocytogenes*), a Gram-positive pathogen, poses a public health risk because of its capacity to persist in refrigerated food chains and withstand extreme stressors. Temperature-dependent motility enhances environmental dissemination. Its clinical manifestations range from self-limiting gastroenteritis to life-threatening sepsis and meningitis. The mortality rate reaches 20%–30% among immunocompromised individuals, and this pathogen also causes fetal loss during pregnancy. While industrialized nations monitor *L. monocytogenes* in processed dairy and meat products, China’s evolving dietary patterns – increasing reliance on cold-stored food demands enhanced surveillance. Elucidating *L. monocytogenes*’s molecular adaptation mechanisms is imperative for mitigating its dual burden on food safety and clinical morbidity [1–4].

*L. monocytogenes* employs two-component systems (TCSs), ubiquitous bacterial signaling systems, comprising histidine kinases (HKs) and response regulators (RRs), to resist environmental challenges, such as acidic, refrigeration temperature, high salt, oxidative stress, and alkaline disinfectants [1–5]. HKs sense extracellular stimuli through their transmembrane sensors and then undergo autophosphorylation at conserved cytoplasmic domains to activate downstream signaling [6,7]. RRs then relay phosphate groups to effector domains, thereby driving transcriptional or post-transcriptional adaptations [8,9]. This design enables TCSs to coordinate virulence expression with stress resilience, such as acid, osmotic, and antibiotic tolerance. Metabolic shifts are critical for *L. monocytogenes*’s dual lifestyle: persistence in refrigerated food and invading host cells [10]. By integrating environmental sensing with virulence regulation, TCSs underpin *L. monocytogenes*’s success as a foodborne pathogen, bridging ecological survival and clinical pathogenesis.

The LiaS two-component system, an evolutionarily conserved intramembrane histidine kinase complex in *L. monocytogenes*, governs virulence and stress adaptation through precise signal transduction. LiaS features dual transmembrane helices and a truncated cytoplasmic domain, whose basal activity is repressed by its membrane partner LiaF [2]. Environmental stressors activate LiaS autophosphorylation, triggering a LiaR-dependent transcriptional cascade that upregulates membrane stabilization, cell wall repair, and cross-resistance to antibiotics, preservatives, and physicochemical extremes, such as pH fluctuation, osmotic stress, and oxidative stress [11,12]. Post-transcriptional fine-tuning occurs via small RNA-mediated regulation of *liaS* mRNA stability. Homologous systems in *Firmicutes* mediate niche-specific adaptations, including lipid II antibiotic resistance (*Bacillus subtilis)*, acid and thermal tolerance (*Staphylococcus aureus)*, bile resistance (*Enterococcus faecium*), and acidic biofilm formation (*Streptococcus pyogenes)* [13–16]. In *L. monocytogenes*, LiaSR (Lmo1021-Lmo1022) critically enables survival in hostile host environments and food-processing environments. Single-residue mutations in LiaS or deletion of *liaS* alter virulence expression and antimicrobial persistence, highlighting its dual roles in pathogenesis and environmental stress [11,17–19]. This study aimed to systematically explore the roles of LiaS in bacterial tolerance to acid, alkali, osmotic pressure, oxidative stress, and heavy metals, as well as in bacterial pathogenesis. This would further explore the molecular mechanism of LiaS and provide strategies for infectious diseases, understanding host-pathogen interactions, and developing antimicrobial development.

## Materials and methods

### Bacterial strains and vectors

*L. monocytogenes* wild-type strains EGD-e, the *liaS* deletion mutant strain (Δ*liaS)* and the complemented Δ*liaS* strain (CΔ*liaS*) were constructed and maintained in our laboratory. Genetic manipulations were performed using the temperature-sensitive shuttle plasmid pKSV7 and the integrative plasmid pIMK2, with *Escherichia coli* DH5α serving as the cloning host. Bacterial cultures were grown in 7 mL Brain Heart Infusion (BHI) broth (Oxoid, Hampshire, England, Cat. No. CM1135B) or 7 mL Luria–Bertani (LB) (Oxoid, Hampshire, England, Cat. No.12780085) medium at 37 °C with aeration. All primers used in this study were designed and listed in Table 1. All reagents in our lab were sourced from Sangon Biotech (Shanghai, China), Merck, or Sigma–Aldrich (St. Louis, MO, USA) and were the highest purity available.

**Table 1.** Primers used in this study.

Primer	Sequence (5`→3`)	Primer purpose
*liaS*-upstream-*Kpn* I-fwd	CGGGGTACCAAAGTTGGGCTTGTTGTTGCAG	Amplify the upstream homologous arm of *liaS* by PCR
*liaS*-upstream-rev	CGCTTTCCACGTCTTTCGAAAAACTCATAGCCTAATCACCTCTAAAT	
*liaS*-downstream-fwd	GAGTTTTTCGAAAGACGTGGAAAGCGAATGATAAAAGTATTACTTGTAGAT	Amplify the downstream homologous arm of *liaS* by PCR
*liaS*-downstream-*Pst* I-rev	AAAACTGCAGTTCTGCGATTAAAAGTAAGATTTCATTTTCACGA	
*liaS*-OSC-*Sac* I-fwd	CGAGCTCAGTAGTAGGCTATGTTCCAACTAGC	Amplify *liaS* locus by PCR
*liaS*-OSC-RH-rev	CCATCATCAGTTTCGAAAAACTCATGCTGACACACCTCCTTACGTTCCTC	
*liaS*-OSC-RH-fwd	GAGGAACGTAAGGAGGTGTGTCAGCATGAGTTTTTCGAAACTGATGATGG	Amplify *liaS* locus by PCR
*liaS*-OSC-*Bam* HI-rev	CGCGGATCCTCATTCGCTTTCCACGTCCTTTTTCG	
In1	TTCCCTTTGTGGTTTTCGTTCC	Verify the deletion strain and complementation strain of *liaS* gene by PCR
In2	CACGCATCTCTGATTGGGATTC	
Out1	ATTATGATTCGTAGCGTTTCGG	Verify the deletion strain and complementation strain of *liaS* gene by PCR
Out2	TTTTGCTTCTTTCCAGTTTTGC	
*BetL*-qPCR-F	TTGGTGTAATCGCTTCCC	Detect *BetL* gene by qPCR
*BetL*-qPCR-R	CCTGTTCTGTCGCTAAACTTG	
*opucD*-qPCR-R	CCCATTGCCTTACCAGATTCT	Detect *opucD* gene by qPCR
*opucD*-qPCR-F	AGTCGTCTTGTCCTTGTTCCT	
*opucC*-qPCR-F	GGTTTTGAAGAGCGTTTCCAC	Detect *opucC* gene by qPCR
*opucC*-qPCR-R	ATTATCCACACCAGCAGTGAG	
*opucB*-qPCR-F	AATTCGTCATTCCCATGCCA	Detect *opucB* gene by qPCR
*opucB*-qPCR-R	AAACCGTTCCGTCTCTTGC	
*opucA*-qPCR-F	CGATACGGTCTGCCAGTTT	Detect *opucA* gene by qPCR
*opucA*-qPCR-R	TACTCGTGATTCCCTACAAGAAGA	
*gbuA*-qPCR-F	GCGTGATGGTTCTGTCGTT	Detect *gbuA* gene by qPCR
*gbuA*-qPCR-R	CAATCATAACATTGCTCGCTG	
*gbuB*-qPCR-F	CAAACTATGCCAGCCTTCGT	Detect *gbuB* gene by qPCR
*gbuB*-qPCR-R	GGCATTGCGAAAATAACAGAAGC	
*gbuC*-qPCR-F	AGAAGATAAACCTTCCGCGTAC	Detect *gbuC* gene by qPCR
*gbuC*-qPCR-R	TTCCACTCCATCGGTCCATT	

### Cell lines

RAW264.7, Caco-2, and L929 cells were purchased from the Wuhan Institute of Virology (Wuhan, China) and maintained in our laboratory. Cells were cultured in Dulbecco’s modified Eagle’s medium (DMEM, Gibco, Cat. No. C11995500BT) or RPMI-1640 medium (Gibco, Cat. No. C11875500BT) supplemented with 10% fetal bovine serum (FBS, Gibco, Cat. No. A5669701) at 37 °C with 5% CO_2_.

### Collection of bone marrow-derived macrophages (BMDMs)

A C57BL/6 mouse (male, 7–8 w, 20–25 g) was euthanized via carbon dioxide asphyxiation (40% cage volume per minute) and the external areas of bodies were disinfected with 5 mL of 70% ethanol (Aladdin, China, Cat. No. R433196-4). The hind limbs were dissected with sterile blunt scissors to expose muscle tissues. Hind legs just above the pelvis were cut with sharp and sterile dissecting scissors, ensuring that the epiphysis remained intact. Hind legs above the claws were cut to remove the lower portion of the hind leg and were transferred to 10 mL of RPMI-1640 medium in a sterile dish for 5 min to loosen the muscle tissue. The hind leg just below the knee joint was cut through the ligaments to remove the tibia and remain epiphysis intact. Any extra muscle tissue on the femur was removed using lint-free tissue paper and gently cleaned the bones with 8 mL of 70% ethanol. The bones were transferred to 10 mL of Hank’s Balanced Salt Solution (HBSS, Solaibio, China, Cat. No. H1147) in a sterile dish for rinsing off ethanol and then transferred to 10 mL of RPMI-1640 medium in a sterile dish. Both ends of the femur and tibia were trimmed with sterile scissors to expose the marrow cavity. Bone marrow was flushed out using a 1 mL insulin syringe equipped with a 29 G × 1/2 inch needle, filled with 2mL of sterile HBSS per bone. The flushed contents were collected into a sterile 50mL conical centrifuge tube.

The cell suspension was passed through a 70-μm cell strainer to remove bone fragments and debris. Cells were centrifuged at 300×g for 3 min. The supernatant was discarded, and the pellet was resuspended in 8 mL of complete medium (RPMI-1640 supplemented with 10% FBS and 1% penicillin/streptomycin; Gibco, Cat. No. 15,140,122). Cells were seeded into 10-cm culture dishes (10 mL BMDMs medium/dish: RPMI-1640 with 10% FBS and 20% L929-conditioned medium as M-CSF source). The cells were evenly distributed by gentle swirling and incubated at 37 °C, 5% CO_2_ for 72 h. Each dish was supplemented with 3 mL of fresh BMDMs medium on Day 3. The cell culture was continued for 7 additional days, 50% medium was replaced every 48 h. Adherent BMDMs were harvested by gentle scraping into 3 mL of cold phosphate‑buffered saline (10 mM PBS, pH 7.4, Amizona Scientific, China, Cat. No. ABS40003-500C) with 60 μL of 100 mM ethylenediaminetetraacetic acid (EDTA) [20] (Amizona Scientific, China, Cat. No. IE9030).

### Experimental animals

Institute of Cancer Research (ICR) mice (female, 6–8 weeks) were obtained from Zhejiang Academy of Medical Sciences. All experimental procedures were conducted in compliance with China’s Regulations for the Administration of Affairs Concerning Experimental Animals and were approved by the Zhejiang Provincial Science and Technology Department’s Institutional Animal Care and Use Committee (Permit Number: ZAFUAC2022033). ICR mice were housed under controlled conditions at 21–26 °C, 50%–60% relative humidity. The animal room maintained a 12-h light/dark cycle at an intensity of 15–20 lux. Mice had free access to drinking water and standard feed (Xietong, China), which was replenished 3–4 times per week. Corn cob (Xietong, China) was used as bedding. After adapting to the environment for 3 d, mice were used for subsequent experiments. All the mice were euthanized by asphyxiation with 40% CO_2_ (cage volume/min) according to the American Veterinary Medical Association after the experiments finished, with death verified prior to disposal. Animal carcasses were sealed in biohazard bags and stored in a −20 °C freezer, and subsequently incinerated by a professional waste management company.

This study was conducted in accordance with the ARRIVE guidelines (Animal Research: Reporting of *n Vivo* Experiments). All animal experiments were reported following the ARRIVE guidelines 2.0: author checklist to ensure transparency and reproducibility.

### Sequence retrieval and primer design

To construct the deletion strain of the *liaS* gene, the sequence of *liaS* gene (lmo1021) along with the five flanking genes was downloaded using the genome sequence of the *L. monocytogenes* standard strain EGD-e (GenBank sequence number NC_003210.1) as a template from the National Center for Biotechnology Information (NCBI) (http://www.ncbi.nlm.nih.gov/). The primers *liaS*-upstream-*Kpn* I-fwd/*liaS*-upstream-rev and *liaS*-downstream-fwd/*liaS*-downstream-*Pst* I-rev were designed using Snapgene software (version 6.0.2) to amplify the upstream homologous arm and the downstream homologous arm, respectively. In addition, for the construction of the complemented strain of the *liaS* gene, the promoter regions flanking the *liaS* gene were predicted and identified using the bioinformatics website (www.biocyc.org). The primers of *liaS*-OSC-*Sac* I-fwd/*liaS*-OSC-RH-rev and *liaS*-OSC-RH-fwd/*liaS*-OSC-*Bam* HI-rev were designed by SnapGene software (version 6.0.2) to amplify the promoter region of *liaS*.

### *liaS* gene in-frame deletion

The mutant construction strategy was performed as described previously [20]. For the construction of the *liaS* gene deleted strain, briefly, the upstream and downstream regions of the *liaS* gene were amplified and assembled via overlap polymerase chain reaction (PCR) to generate the target fragment, and then ligated into the temperature-sensitive shutttle plasmid pKSV7. pKSV7 plasmids were electroporated into competent EGD-e cells. The transformants were grown in 7 mL of BHI at 42 °C with 7 μL of chloramphenicol (10 mg/mL, Sangon Biotech, China, Cat. No. A429048-0005) to knock out the target gene after successful exchange, the bacteria were passaged at 30 °C in 7 mL of chloramphenicol-free medium to remove the pKSV7 plasmid. Successful mutants were confirmed by diagnostic PCR using the flanking primers (In1/In2) and locus-specific primers (Out1/Out2). According to the manufacturer’s instructions (Toroivd, Shanghai, China), the PCR reaction system consists of 2 μL template, 1.5 μL each of forward and reverse primers, 25 μL of 2×TOROBlue® Flash KOD Dye Mix, and 20 μL of ddH_2_O, making a total volume of 50 μL. The PCR reaction procedure was carried out using the following procedure: initial pre-denaturation at 98 °C for 3 min; followed by 35 cycles of denaturation at 98 °C for 10 sec, annealing at 60 °C for 5 sec, and extension at 68 °C for 5 sec and a final extension step at 68 °C for 5 min to ensure complete elongation of all PCR amplicons.

### Complementation strain construction of liaS gene deletion mutant

CΔ*liaS* was generated by chromosomal integration of the *liaS* open reading frame (ORF) with its native promoter into the Δ*liaS* mutant using the integrative plasmid pIMK2. The *liaS* locus was amplified using the primers *liaS*-OSC-RH-fwd/*liaS*-OSC-*Bam* HI-rev and *liaS*-OSC-*Sac* I-fwd/*liaS*-OSC-RH-rev, cloned into pIMK2 via *Sac* I/*Bam* HI digestion to replace the Phelp promoter, and electroporated into the Δ*liaS* strain. Transformants were selected on BHI agar containing 50 μg/mL of kanamycin (Sangon Biotech, China, Cat. No. A463711-0500). Successful complementation was confirmed through PCR verification using the primers In1/In2 and Out1/Out2, ensuring restored *liaS* expression under endogenous regulatory control.

### Growth kinetics analysis under different stress conditions

*L. monocytogenes* can tolerate various harsh environments, such as acidic, alkaline, osmotic, and oxidative stresses [21]. The growth kinetics of *L. monocytogenes* strains (EGD-e, Δ*liaS,* and CΔ*liaS*) were assessed under different stress conditions. Overnight cultures in 7 mL BHI (37 °C, 200 rpm) were harvested (5,000×g, 5 min), washed with 10 mL PBS (pH 7.4), and standardized to OD_600 nm_ of 0.6. Bacterial suspensions were diluted 1:50 in fresh BHI adjusted to the following defined stressors: pH 5.0 (acidic), pH 9.5 (alkaline), or hyperosmotic condition (7% NaCl) (Sangon Biotech, Shanghai, China, Cat. No. A610476-0005). Cultures were incubated for 13 h (37 °C, 200 rpm), and the OD_600 nm_ recorded hourly. Viable cell counts were determined at 4, 6, and 8 h by serial dilution plating on BHI agar. Bacterial suspensions were exposed to at pH 3.0 lactic acid for 1 h at 37 °C. Viability was assessed by serial dilution plating on BHI agar, and colonies were enumerated after incubation for 24 h. Bacterial growth curves were plotted using GraphPad software 8.0.2. The experiment was repeated three times.

### Gene expression analysis by quantitative real-time PCR

Expression levels of stress-related genes (*BetL*, *OpucA-D*, *gbuA-C*) in *L. monocytogenes* strains were determined by qPCR. Overnight cultures grown in 7 mL BHI broth (37 °C, 200 rpm) were harvested (5,000 ×g, 5 min), washed twice with 10 mL cold PBS (pH 7.4), and adjusted to an OD_600 nm_ of 0.6. Bacterial suspensions were exposed to pH 3.0 with lactic acid for 1 h at 37 °C. Total RNA was extracted using 1 mL of TRIzol reagent (Thermo Fisher Scientific, Waltham, USA, Cat. No. 15596018CN). RNA concentration and purity were assessed using a NanoDrop 2000 spectrophotometer. The RNA concentration was higher than 300 ng/μL and OD_260 nm/280 nm_ was 1.8–2.0. cDNA was synthesized from 1 µg of total RNA using the HiScript III All-in-one RT SuperMix Perfect for qPCR (Vazyme, Wuhan, China). The reverse‑transcription reaction consisted of 1 µL of Enzyme mix and 4 µL of 5× All-in-one RT SuperMix, and ddH_2_O was added to bring the total volume to 20 µL. The reaction was incubated at 50 °C for 15 min and 85 °C for 5 sec. qPCR was performed using SYBR Green (Vazyme, Wuhan, China) on a real-time PCR detection system (Agilent Technologies, Santa Clara, USA). The reaction mixture was prepared as follows: 2× SYBR Green qPCR Master Mix 10 µL, 0.4 µL of forward primer (10 µM), 0.4 µL of reverse primer (10 µM), template cDNA 20 ng and ddH_2_O to a final volume of 20 µL. The thermal cycling protocol consisted of three stages: Initial denaturation stage (95 °C for 30 sec); Amplification cycles stage (95 °C for 10 sec, 60 °C for 30 sec for 35 cycles); Melt curve analysis stage (95 °C for 15 sec, 60 °C for 60 sec, 95 °C for 15 sec) to confirm amplification specificity and avoid nonspecific products. 16S rRNA was used as the internal reference gene. The relative gene expression levels were calculated using the 2^–ΔΔCt^ method. All experiments were conducted in triplicate.

### Oxidative stress tolerance assay

The wild-type EGD-e, Δ*liaS,* and CΔ*liaS* were each added to the BHI medium at a final concentration of 20 mM H_2_O_2_ (Merck, Cat. No. 323,381) and incubated at 37 °C for 2 h. The bacterial solution before and after the effect of H_2_O_2_ was serially diluted, and the plates were spotted and incubated overnight at 37 °C, and the growth rate was calculated from the colony count.

H_2_O_2_ susceptibility assay of *L. monocytogenes* strains (EGD-e, Δ*liaS,* and CΔ*liaS*) was assessed by exposing mid-log-phase cultures to 20 mM H_2_O_2_ in BHI broth at 37 °C for 2 h. Pre- and post-treatment bacterial suspensions were serially diluted (10-fold) in 10 mM PBS and plated on BHI agar. Following overnight incubation at 37 °C, colony-forming units (CFU) were enumerated. The growth rate was calculated.

The oxidative stress susceptibility of *L. monocytogenes* strains was evaluated using diamide and metal ions (Cu^2+^/Cd^2+^). Overnight BHI cultures were adjusted to an OD_600 nm_ of 0.6 in PBS. Bacterial suspensions were serially diluted (6-fold, 10 μL/dilution) and spotted onto BHI agar plates supplemented with gradient concentrations of the following stressors: 2.0 and 2.5 mM of diamide (Sangon Biotech, China, Cat. No. A414167-0010), 0.5, 1.0, and 1.5 mM copper chloride (Merck, Cat. No. 451,665) or 0.5, 1.0, and 1.5 mM cadmium chloride (Merck, Cat. No. 202,908). Plates were incubated at 37 °C for 24–48 h, and growth inhibition was quantified by colony viability analysis.

### Bacterial motility and flagellar observation

Motility phenotypes of *L. monocytogenes* strains were assessed using 0.25% soft tryptone soy agar (TSA, Oxoid, Hampshire, England, Cat. No. CM0131B). Overnight cultures were adjusted to an OD_600 nm_ of 0.6 and then inoculated onto agar plates with sterile toothpicks at 30 °C or 37 °C for 48 h. Motility was assessed by measuring the radius from the center of inoculation to the outer edge of bacterial spreading. For flagellar visualization, bacteria grown at 30 °C were adsorbed onto formvar‑coated copper grids for 3 min, negatively stained with 2% phosphotungstic acid (pH 4.5, 10 s), and imaged using transmission electron microscopy (TEM). Flagellar number and morphology were analyzed using the ImageJ and GraphPad software (Version 8.0.2).

### Adhesion and invasion assay in Caco-2 cell

To assess bacterial pathogenicity, *L. monocytogenes* strains were cultured to an OD_600 nm_ of 0.6. Bacteria were serially diluted in PBS (10 mM, pH 7.4), then resuspended in 1 mL RPMI-1640 and used to infected Caco-2 cells at a Multiplicity of infection (MOI) of 10:1. Adhesion was quantified 30 min after washing with 1 mL PBS, 500 µL trypsin-EDTA (0.25%) (Gibco, Cat. No. 25,200–072) lysis, and CFU enumeration. For invasion, cells were incubated in 1 mL fresh medium containing 100 μg/mL gentamicin (Sangon Biotech, China, Cat. No. A462549) for 1 h after the same 0.5 h infection and 1 mL PBS washing, to kill all extracellular bacteria. Cells were then lysed and plated to quantify intracellular bacteria. Intracellular proliferation was analyzed after 12 h, followed by bacterial recovery. This integrated workflow enables the systematic evaluation of bacterial-host interaction dynamics across key infection stages.

### Intercellular growth in murine RAW264.7 macrophages and BMDMs

The intracellular proliferation of *L. monocytogenes* in RAW264.7 macrophages was evaluated as follows: macrophage monolayers in 1 mL of DMEM plus 10% FBS were infected at an MOI of 1:10 for 1 h. Extracellular bacteria were removed by washing with 1 mL PBS, and intracellular survival was enabled by 50 μg/mL gentamicin treatment 1 h post-infection. The medium was replaced with 0.5 mL DMEM supplemented with 10% FBS and 5 μg/mL gentamicin for residual bacterial suppression. At 2, 5, 8, and 12 h post-infection, triplicate wells were lysed with ice-cold sterile water (1 mL/well). Lysates were serially diluted (10-fold) and plated on BHI agar for CFU enumeration. Parallel assays in BMDMs followed identical protocols, with cell-specific validation of bacterial proliferation kinetics.

### Plaque assay in L929 fibroblasts

Plaque assay was performed using conventional methods. Briefly, mouse L929 fibroblasts were maintained in 5 mL high-glucose DMEM supplemented with 10% FBS and 2 mM L-glutamine (Gibco, Cat. No. 25,030,081). Cells were plated at 1 × 10^6^ cells/well in a 6-well dish (1 mL/well) and infected at an MOI of 1:5 with *L. monocytogenes* at 37 °C with 5% CO_2_ for 1 h. Extracellular bacteria were killed with 50 μg/mL gentamicin for an additional 1 h, and the cells were washed three times with 2 mL PBS (10 mM) and then overlaid with 3 mL of medium (10% FBS) plus 0.7% agarose (Sangon Biotech, China, Cat. No. A620014) and 10 μg/mL gentamicin. Following a 72 h incubation at 37 °C, cells were fixed with 1 mL paraformaldehyde (4%, Sangon Biotech, China, Cat. No. E672002-0500) for 1 h and stained with 1 mL per well of 0.5% (w/v) crystal violet (Micklin, China, Cat. No. C805211). The diameter of the plaques was measured using ImageJ software. Statistical analysis was performed using GraphPad Prism software (Version 8.0.2). The experiments were repeated three times.

### Virulence assay in mouse model

To evaluate the effect of LiaS on bacterial colonization in mice, 8 mice per group were infected with EGD-e, Δ*liaS,* and CΔ*liaS* by intraperitoneal injection (200 µL, 1 × 10^7^ CFU/mL). For bacterial burden analysis, data were analyzed with Log-rank (Mantel-Cox) test, and survival rates were plotted for 6 d using GraphPad software (Version 8.0.2). The livers and spleens were aseptically harvested, homogenized in 2 mL PBS (pH 7.4), serially diluted, and plated on BHI agar for CFU enumeration after 24 h and 48 h of incubation. The statistical difference was analyzed by two-way ANOVA, and survival rates were plotted using GraphPad software (Version 8.0.2).

### Statistical analysis

Statistical analyses were performed using GraphPad Prism 8.0.2. All data were obtained from at least three independent biological replicates (*n* ≥ 3) and are presented as mean ± standard deviation (SD). Comparisons between two groups (control/WT vs treatment/mutant/complemented) were analyzed by unpaired Student’s t-test. For multi-group comparisons, one-way ANOVA was adopted with strain as the factor; for time-dependent assays, two-way ANOVA was used to analyze the effects of strain, time, and their interaction. Tukey’s test was used for post-hoc pairwise comparisons after ANOVA. Normality (Shapiro–Wilk test) and variance homogeneity (Bartlett’s test) were examined prior to analysis, and non-parametric tests (Kruskal–Wallis with Dunn’s test) were used if assumptions were not met. *p* values were denoted as follows: ns (*p* > 0.05), *(*p* < 0.05), ** (*p* < 0.01), *** (*p* < 0.001), **** (*p* < 0.0001).

## Results

### LiaS is essential for *L. monocytogenes* to resist pH, osmotic, and oxidative stresses

The *liaS* gene deletion and complementation strains were constructed using EGD-e as the backbone. The PCR analysis confirmed successful strain construction, with amplification products matching the expected fragment sizes. For the *liaS* deletion mutant, primers In1/In2 produced no amplification, while flanking primers Out1/Out2 generated a truncated band consistent with the deleted region. In the complementation strain, both primer pairs yielded bands of predicted sizes, validating precise gene deletion and complementation (Figure S1).

**Figure 1.**
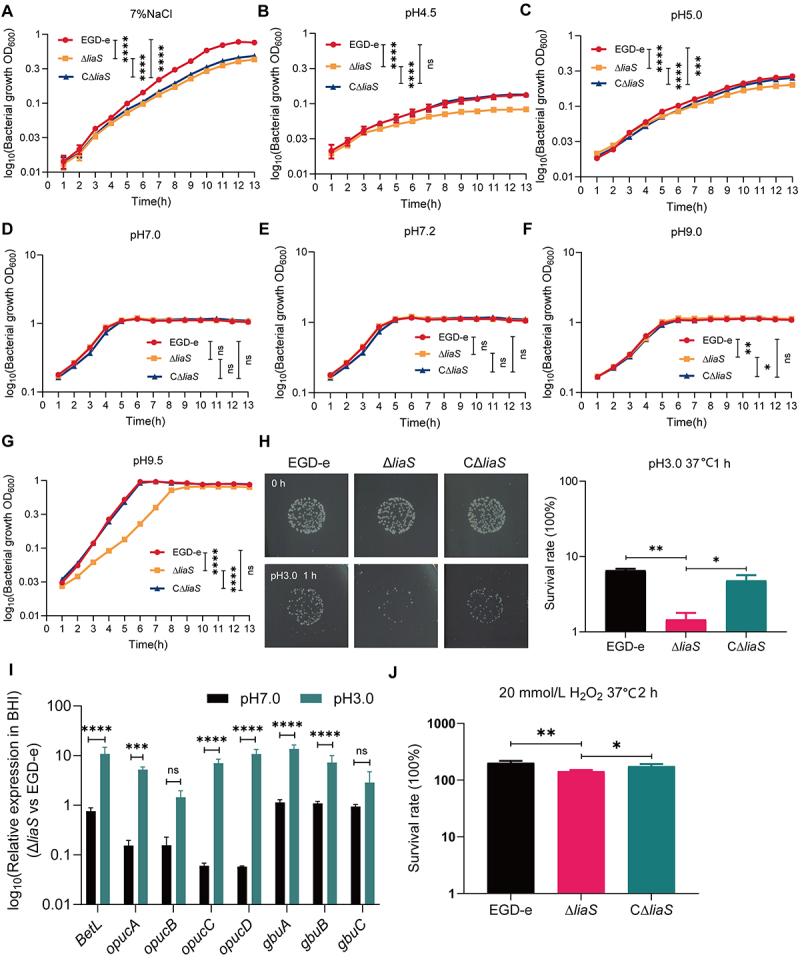
LiaS is essential for *L. monocytogenes* in responding to hostile environmental elements. Growth curves and bacterial loads of the EGD-e, Δ*liaS* and CΔ*liaS* strains under 7% NaCl (A), pH 4.5 (B), pH 5.0 (C), pH 7.0 (D), pH 7.2 (E), pH 9.0 (F), pH 9.5 (G), and pH 3.0 (H). Relative expression levels of osmotic stress-related genes (*BetL, opucA*, *opucB, opucC*, *opucD, gbuA, gbuB,* and *gbuC)* in Δ*liaS* compared to the EGD-e under neutral and acid stress conditions (I). Growth curves and bacterial loads of the EGD-e, Δ*liaS,* and CΔ*liaS* strains under 20 mmol/L H_2_O_2_ (J). Data are presented as mean ± SD from three independent experiments (A–J). *means *p* < 0.05, ** means *p* < 0.01, *** means *p* < 0.001, **** means *p* < 0.0001, ns means no significance.

The growth capacity and bacterial loads of wild-type EGD-e, Δ*liaS*, and CΔ*liaS* strains were evaluated under the following stress conditions: 7% sodium chloride, pH 3.0, pH 4.5, pH 5.0, pH 7.0, pH 7.2, pH 9.0, and pH 9.5. Under 7% NaCl-induced osmotic stress, bacterial growth of the Δ*liaS* strain was significantly inhibited compared with the wild-type EGD-e (*p* < 0.0001) and this growth defect was fully rescued in the CΔ*liaS* strain (*p* < 0.0001) (Figure 1(A)). Bacterial growth was significantly reduced in the Δ*liaS* strain compared to the wild-type strain (*p* < 0.0001), and completely restored in the CΔ*liaS* strain upon acidic conditions (*p* < 0.0001) (Figure 1(B,C)), and at pH 9.5 (*p* < 0.0001) (Figure 1(G)). The results showed no significant differences in growth capacity among the three strains under neutral (pH 7.0 and pH 7.2) (Figure 1(D,E)). The growth capacity was reduced in the Δ*liaS* strain compared to the wild-type strain (*p* < 0.01) and restored in the CΔ*liaS* strains at mildly alkaline conditions (pH 9.0) (*p* < 0.05) (Figure 1(F)). To further assess acid‑stress tolerance, a survival assay under strong acid (pH 3.0) revealed that the Δ*liaS* strain had a markedly lower survival rate than the wild-type (*p* < 0.01), and partially restored compared to the CΔ*liaS* strains (*p* < 0.05) (Figure 1(H)). In addition, compared with neutral conditions, the expression levels of osmotic stress-related genes (*BetL*, *opucA*, *opuc*C, *opucD,* and *gbuA-B*) were markedly upregulated in Δ*liaS* compared to EGD-e in acidic condition (*p* < 0.001), suggesting that LiaS is involved in resisting acidic stress by regulating gene transcriptional expression (Figure 1(I)). The amplification curves for all qPCR assays showed consistent exponential fluorescence increase with no nonspecific amplification, confirming the reliability of the expression data (Figure S2).

To investigate whether LiaS mediates oxidative stress adaptation, bacterial growth was assessed under oxidative conditions induced by hydrogen peroxide (20 mM H_2_O_2_). The Δ*liaS* strain showed significantly reduced proliferation under hydrogen peroxide (*p* < 0.01), whereas the CΔ*liaS* strain restored growth to wild-type levels under these conditions (*p* < 0.05) (Figure 1(J)). These results indicate that LiaS is essential for *L. monocytogenes* growth under oxidative stress conditions.

### LiaS is sensitive to copper ions

**Figure 2.**
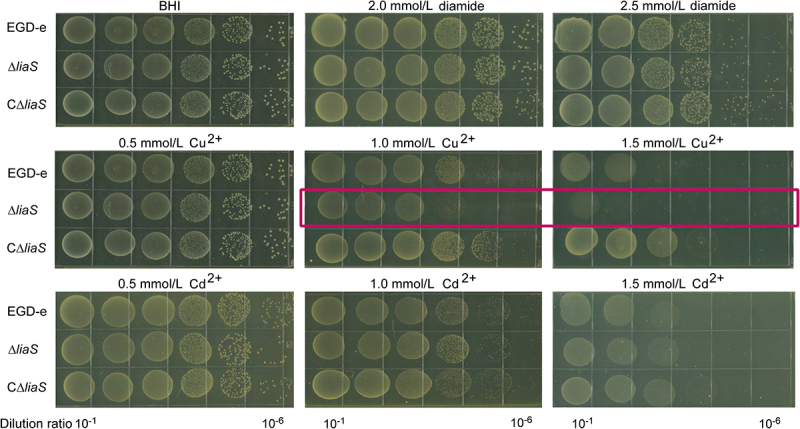
LiaS is sensitive to copper ion. Growth capacity of the EGD-e, Δ*liaS,* and CΔ*liaS* strains under 2.0 mM or 2.5 mM diamide, 0.5 mM, 1.0 mM, or1.5 mM CuCl_2_, 0.5 mM, 1.0 mM, or1.5 mM CdCl_2_. The resistance of the Δ*liaS* was decreased 6-fold to EGD-e under 1.0 mM CuCl_2_ or 1.5 mM CuCl_2_. While resistance of Δ*liaS* to other oxidative substances, diamide, CdCl_2_, showed no difference.

To investigate whether LiaS mediates the response to heavy metal stress, we assessed bacterial growth under conditions induced by different heavy metals, including 0.5, 1.0, and 1.5 mM Cu^2+^, 2.0 and 2.5 mM diamide and 0.5, 1.0, and 1.5 mM Cd^2+^. The results showed no significant differences in growth among the EGD-e, Δ*liaS*, and CΔ*liaS* strains under diamide or Cd^2+^. However, under copper ion stress (1.0 and 1.5 mM Cu^2+^), the Δ*liaS* strain exhibited significantly reduced bacterial proliferation compared to the wild-type, whereas the complementation strain restored growth comparable to EGD-e (Figure 2). These findings indicated that LiaS plays a crucial role in the resistance of *L. monocytogenes* to copper-induced stress.

### LiaS is vital for the motility of *L. monocytogenes*

**Figure 3.**
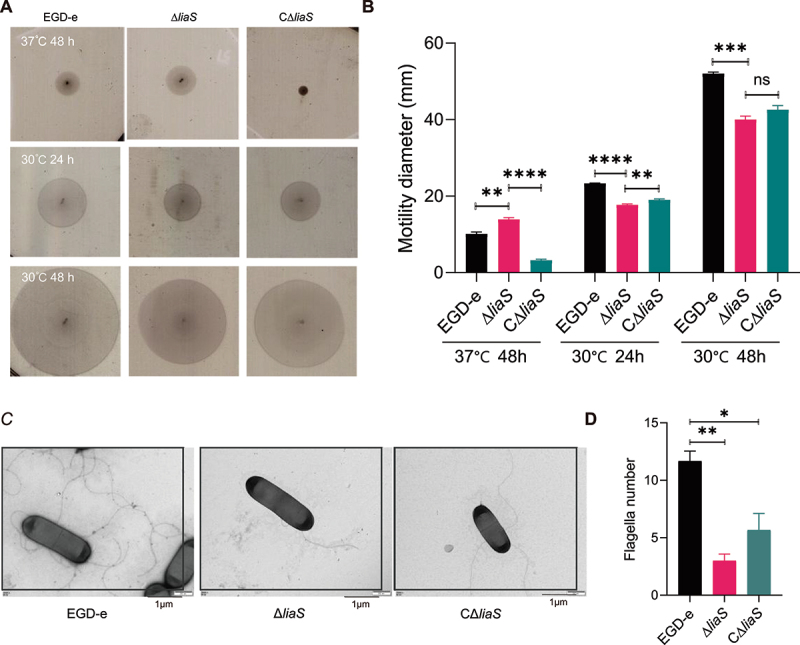
LiaS is also essential for the motility of *L. monocytogenes*. Motility assay of the EGD-e, Δ*liaS* and CΔ*liaS* strains at 30 °C and 37 °C on semi-solid media (A–B); flagella observation of bacterial flagella at 30 °C after 16-h cultivation (C); and the flagella number was analyzed in three different fields (D). Data are presented as mean ± SD from three independent experiments (A–D). *means *p* < 0.05, ** means *p* < 0.01, *** means *p* < 0.001, **** means *p* < 0.0001, ns means no significance.

To further clarify whether LiaS is involved in the motility of *L. monocytogenes*, bacterial motility on semi-solid media and flagella number were observed. The results showed that at 37 °C, the range of bacterial motility was larger in the Δ*liaS* strain compared to the EGD-e (*p* < 0.01) (Figure 3(A,B)). The diameter of motility of the *liaS* deletion strain was significantly lower than that of the wild-type at 30 °C for 24 h (*p* < 0.0001) and 48 h (*p* < 0.001), the motility ability of the complementation strain was partially restored at 24 h (*p* < 0.01) and 48 h, but did not fully recover to the level of the wild-type strain (Figure 3(A,B)). Additionally, it was observed that the flagellar number of the Δ*liaS* strain was significantly lower than that of the wild-type strain, whereas the complemented strain showed an increase. The number of flagella per field of view: EGD-e strain: 10, Δ*liaS* strain: 2, CΔ*liaS* strain: 3, respectively (Figure 3(C)), the flagella number was analyzed in three different fields of view, the flagella number was significantly reduced in the Δ*liaS* strain compared to the EGD-e (*p* < 0.01), and partly restored in the CΔ*liaS* strain (*p* < 0.05) (Figure 3(D)). In summary, LiaS is involved in bacterial motility.

### LiaS is mainly involved in the cellular invasion process of *L. monocytogenes*

**Figure 4.**
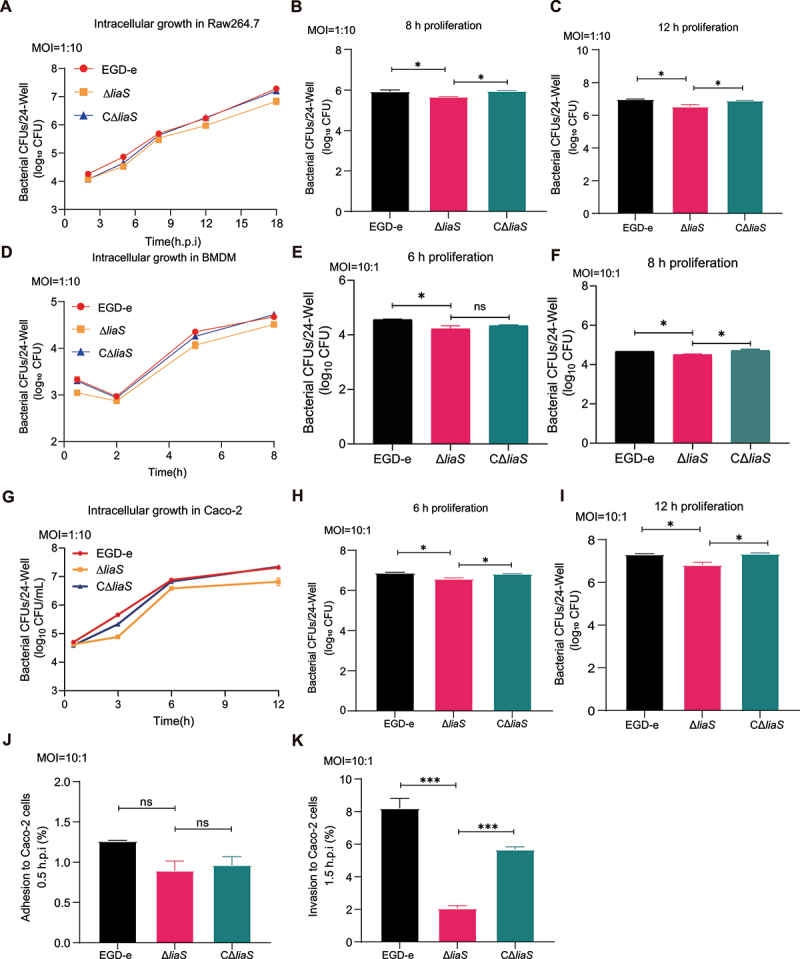
LiaS is mainly involved in the cellular invasion process of *L. monocytogenes*. Intracellular growth of the EGD-e, Δ*liaS,* and CΔ*liaS* strains in RAW264.7 cells (A-C), BMDMs (D–F), and Caco-2 cells (G–I). Adhesion and invasion abilities of the EGD-e, Δ*liaS,* and CΔ*liaS* strains in Caco-2 intestinal epithelial cells (J–K). Data are presented as mean ± SD from three independent experiments (A–K). *means *p* < 0.05, ** means *p* < 0.01, *** means *p* < 0.001, ns means no significance.

Next, we explored whether LiaS is involved in intracellular growth. The proliferation of the Δ*liaS* strain was lower than that of the EGD-e strain and was restored in the CΔ*liaS* strain in RAW264.7 after 8 and 12 h infection (*p* < 0.05) (Figure 4(A–C)). The same trend was observed in BMDMs after 6 and 8 h infection (*p* < 0.05) (Figure 4(D–F)). Additionally, a similar trend was observed in Caco-2 cells (*p* < 0.05) (Figure 4(G–I)). To further elucidate the effects of LiaS on the adhesion and invasion of *L. monocytogenes*, the adhesion and invasion abilities of the EGD-e, Δ*liaS,* and CΔ*liaS* strains in Caco-2 cells were assessed. The results showed that there were no significant differences in bacterial adhesion rates among the wild-type strain, Δ*liaS* mutant and complemented strain after 0.5 h of incubation (Figure 4(J)). However, the invasion rate of the *liaS* deletion strain in Caco-2 cells was significantly lower than that of the wild-type strain and restored in *liaS* complementation strain after incubation with cells for 1.5 h (*p* < 0.001) (Figure 4(K)). These data collectively indicate that LiaS is mainly involved in invasion rather than adhesion of *L. monocytogenes*.

### LiaS is key for *L. monocytogenes* in intercellular migration and pathogenicity

**Figure 5.**
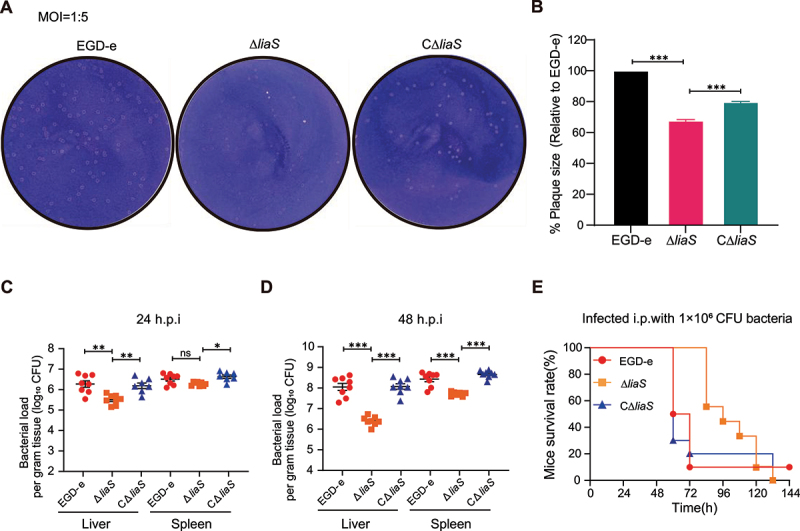
LiaS is key for *L. monocytogenes* to intercellular migration and pathogenicity. Plaque sizes and numbers formed by EGD-e, Δ*liaS,* and CΔ*liaS* strains in L929 fibroblast cell (A). The plaque sizes of EGD-e, Δ*liaS,* and CΔ*liaS* strains were indicated as a percentage (B). Bacterial loads in liver and spleen at 24 h (C) and 48 h (D) post-infection in mice. Mortality was monitored in mice infected with EGD-e, Δ*liaS,* and CΔ*liaS* strains (E). Data are presented as mean ± SD from three independent experiments (A–B). The mean ± SD of eight independent experiments is shown in the data (C–E). *means *p* < 0.05, ** means *p* < 0.01, *** means *p* < 0.001, ns means no significance.

To clarify the role of LiaS in the intercellular migration of *L. monocytogenes*, bacterial virulence was assessed using plaque assays and murine infection models. The migration ability of the EGD-e, Δ*liaS* and CΔ*liaS* strains in L929 cells was tested using plaque assay. The results showed that the diameter of plaques in the Δ*liaS* strain was significantly reduced compared to the wild-type and was restored in the CΔ*liaS* strain after incubation for 1 h (*p* < 0.001) (Figure 5(A,B)), indicating that LiaS impairs the intercellular migration ability of *L. monocytogenes*.

To evaluate the bacterial loads in the livers and spleens of mice infected with the EGD-e, Δ*liaS* and CΔ*liaS* strains, we performed an *in vivo* pathogenicity assay. The results showed that the bacterial loads in the livers of mice infected with Δ*liaS* were significantly lower than those in mice infected with the EGD-e and CΔ*liaS* strains 24 h after intraperitoneal injection (*p* < 0.01) and 48 h (*p* < 0.001) (Figure 5(C,D)). The bacterial loads in the spleens of mice infected with Δ*liaS* was also significantly lower than that in mice infected with the EGD-e and CΔ*liaS* strains after 48 h (*p* < 0.001) (Figure 5(C,D)). Although the survival rate of mice was not effectively reduced by Δ*liaS*, it greatly slowed down the rate of death in the survival assay, indicating that deletion of *liaS* weakens the pathogenicity of *L. monocytogenes* in the early stage of infection (Figure 5(E)). Collectively, the deletion of *liaS* alters intercellular migration and pathogenicity, which indicates that LiaS is not only a stress response factor, but also regulates virulence and pathogenicity for *L. monocytogenes*.

## Discussion

*L. monocytogenes* is a zoonotic pathogen that possesses a remarkable ability to thrive and proliferate under harsh environments frequently encountered in food production facilities [22]. Acidic foods, refrigeration temperatures, high salt, oxidative stress, and alkaline disinfectants are all typical harsh environments that *L. monocytogenes* encounters within the food processing chain [5]. The persistence of *L. monocytogenes* can be attributed to a combination of external factors, including inadequate hygiene practices and ineffective cleaning and sanitation protocols, as well as the presence of specific genomic traits in certain strains of *L. monocytogenes* that enable them to endure over extended periods [23,24].

In bacteria, TCSs enable adaptation to environmental changes via HK-RR pairs [19]. *L. monocytogenes* EGD-e harbors 15 HK/RR pairs and one orphan RR. The key systems include LisRK (Lmo1377/1378) and CesRK (Lmo2422/2421), which are linked to antibiotic tolerance and virulence [7]. AgrAC (Lmo0051/0050), homologous to *Staphylococcus aureus* AgrAC, is critical for quorum sensing and murine virulence [25]. ResDE (Lmo1948/1947) regulates aerobic and anaerobic respiration, whereas PhoPR (Lmo2501/2500) governs phosphate homeostasis [26]. YycFG homolog (Lmo0287/0288) modulates cell wall metabolism and membrane synthesis [27]. KdpED (Lmo2678/2679), analogous to *E. coli* KdpED, activates K^+^ uptake under osmotic stress [28]. In addition, KdpDE and LiaSR contribute to osmotic adaptation [28]. The orphan RR DegU (Lmo2515), which resembles *Bacillus subtilis* DegU, regulates proteases and competence genes [29]. These systems collectively coordinate bacterial survival, virulence, and stress responses, thereby highlighting their roles in pathogenicity and environmental resilience.

The ability to sense acidic stress is vital for the survival and proliferation of microorganisms in harsh environments (Wu et al. 2024). We found that bacterial growth of *L. monocytogenes* was impaired in the *liaS* deleted strain in acidic or alkaline conditions (pH 3.0 or pH 9.5) (Figure 1), particularly at pH 3.0, and it exerted an intense positive effect on acid resistance, which means that LiaS is vital to resist acidic conditions for infection and survival, which is consistent with the study that deletion of *liaS* markedly impaired growth ability of *L. monocytogenes* EGD-e at pH 5.6 [11]. However, the study demonstrated that LiaSR is involved in inhibiting the acid resistance of *L. monocytogenes* 10403S [19] which may be due to the different parental strains used in the two studies. In addition, we further assessed the impact on osmotic pressure, and the results showed that deletion of *liaS* significantly inhibited bacterial growth and load in 7% NaCl (Figure 1). This suggests multiple roles for LiaS in the stress tolerance of *L. monocytogenes*, which is consistent with a study showing that the maximum growth rate of the Δ*liaS* strain decreased by 67% in 6% NaCl [11]. We also clarified the regulation of LiaS in oxidative stress, and the results revealed that deletion of *liaS* significantly inhibited bacterial growth under 20 mM H_2_O_2_ and enhanced bacterial growth in the CΔ*liaS* strains (Figure 1), which further demonstrated that LiaS plays an important role in oxidative stress. Previous studies have confirmed that the growth of *L. monocytogenes* changes slightly in the presence of 5 mM H_2_O_2_, which may be due to the concentration of H_2_O_2_ [11,30]. Besides, LiaS also mediates cellular response in heavy metal condition, the results showed that the growth of the Δ*liaS* strain was significantly decreased compared to the EGD-e under 1.0 mM or 1.5 mM Cu^2+^, particularly in 1.5 mM Cu^2+^ (Figure 2). Researchers have demonstrated that the CopRS of TCSs upregulates both copper resistance and lipoprotein remodeling genes upon copper challenge in *L. monocytogenes* [31,32]. The mechanism of action of LiaS in regulating Cu^2+^ will be further elucidated, Is Cu^2+^ a direct signal molecule for LiaS activation, or is it sensed indirectly? Which key residues are essential for LiaS to sense Cu^2+^ and transduce the signal? We further evaluated the expression of stress-related genes to elucidate the regulatory role of LiaS at the transcriptional level under acidic conditions (pH 3.0), genes other than *opucB* and *gbuC* were significantly upregulated in the Δ*liaS* strain compared to the wild-type, indicating that these genes play a key role in regulating acidic stress, except for those involved in the growth of *L. monocytogenes* (Figure 1). This is consistent with the impaired osmotic tolerance observed phenotypically, and suggests that LiaS negatively regulates these transport systems to maintain membrane stability under hyperosmotic stress [33]. These findings indicate that LiaS is not only involved in environmental sensing but also coordinates the transcription of key stress-adaptive genes, which supports our conclusion that LiaS plays a central role in the regulation of multiple protective mechanisms, contributing to *L. monocytogenes* survival under various hostile conditions. Altogether, LiaS possesses the ability to overcome extreme stress conditions, which is key for *L. monocytogenes* to survive in adverse environments, particularly in acid, alkali, osmotic pressure, oxidative stress, and copper ion. This provides a theoretical basis for the synthesis of histidine kinase inhibitors and facilitates food safety and public health.

Apart from the function of LiaS in the stress response, whether LiaS mediates pathogenesis remains unclear. We found that the motility of the Δ*liaS* strains was significantly decreased at 30 °C for 24 h and 48 h, and the number of flagella was decreased compared to the wild-type EGD-e (Figure 3). We further investigated whether the deletion of *liaS* led to cell envelope changes that affected bacterial attachment and invasion. The results showed that invasion rather than adhesion was significantly reduced in the Δ*liaS* strains and recovered in the CΔ*liaS* strains (Figure 4), indicating that LiaS activation makes the cells more invasive. Studies have demonstrated that LisRK of *L. monocytogenes* is essential for adhesion, invasion, acidic conditions, and some β-lactam antibiotics [32,34]. However, the mechanism by which LiaS mediates bacterial invasion requires further exploration. In addition, LiaS slightly influenced the proliferation of different cell lines, suggesting that it may not be a vital factor in the regulation of *L. monocytogenes* proliferation (Figure 4). Studies have found that the deletion of *liaSR* results in a significantly higher bacterial loads than the wild-type 10403S in different tissues [19]. The intercellular migration of *liaS* deletion strains was significantly inhibited compared to that of the wild type, and was restored in complementation strains in our study (Figure 5). Importantly, the bacterial loads in the livers and spleens were significantly reduced in *liaS* deletion strains compared to the wild type (Figure 5). Mouse survival was delayed in the deletion of *liaS* strains, although the survival rate did not change (Figure 5). At present, five of the TCSs of *L. monocytogenes* have been related to its pathogenicity: CesRK, LisRK, ViRS, LiaSR, and PieRS [19,25]. Our study showed that LiaS is vital for motility, invasion, and pathogenicity, which further enriched the functionalities of TCSs, providing a basis for research on their mechanisms, as well as the design of inhibitors targeting TCSs. Some researchers have identified a wide range of molecules capable of inhibiting HK activity, such as antibiotics [35,36]. HK inhibitor-targeted LiaS will be explored further. In addition, LiaSR has been found to be associated with antiseptic resistance in other bacteria, such as *Streptococcus mutans*, *Bacillus subtilis,* and *Staphylococcus aureus* [37–39]. Hence, we aimed to clarify the role of LiaS in antiseptic resistance and investigate effective antiseptic production against *L. monocytogenes*. The dataset that supports the findings of this study is available on the figshare database [40].

**Figure 6.**
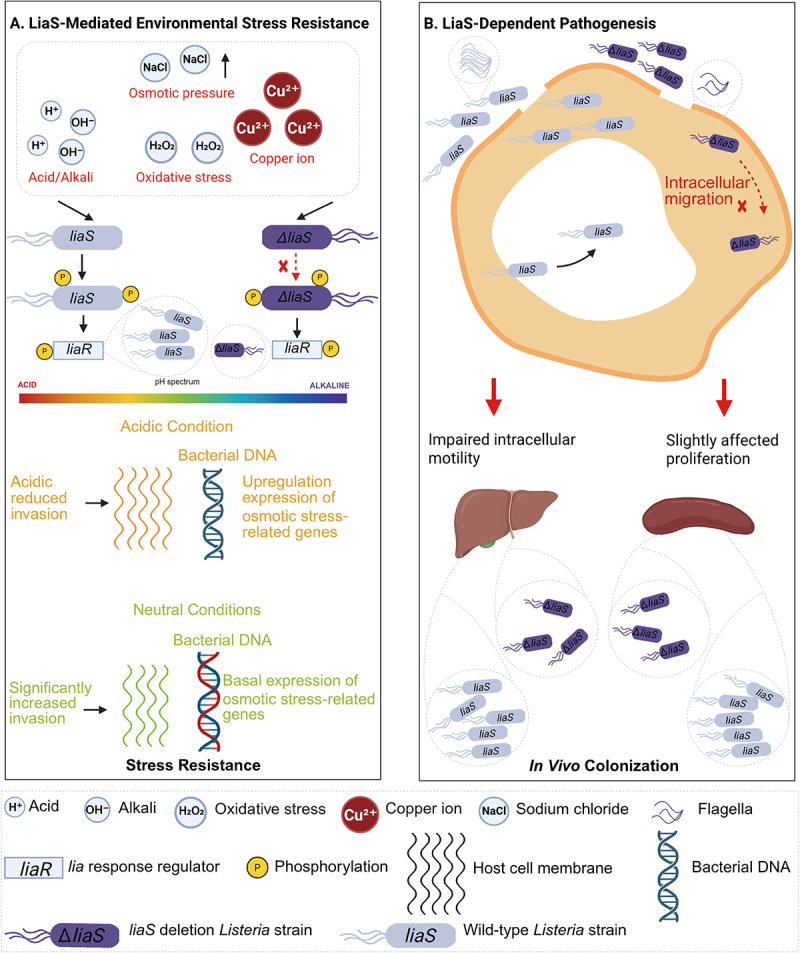
Schematic representation of LiaS system contributing to stress response and pathogenicity in *L. monocytogenes*. Panel A: LiaS‑mediated antagonism of environmental stresses. Under a variety of adverse conditions – including osmotic stress (NaCl), oxidative stress (H_2_O_2_), acidic/alkaline pH (H^+^/OH^−^), and copper ion stress (Cu^2+^) – the sensor kinase LiaS becomes activated and auto-phosphorylated. The phosphoryl group is subsequently transferred to the response regulator LiaR, establishing the LiaS‑LiaR signaling cascade. This cascade modulates bacterial proliferation; deletion of *liaS* leads to reduced bacterial growth under these harsh conditions. Moreover, in acidic environments, genes associated with osmotic stress are up‑regulated, leading to reduced bacterial invasiveness, whereas under neutral pH, these genes maintain basal expression and bacterial invasiveness is markedly enhanced. Panel B: LiaS‑dependent virulence mechanism. Deletion of *liaS* impairs bacterial invasion and intracellular motility. The functional impairment of *liaS* leads to a reduction in flagellar number and compromised intracellular movement, which in turn decreases colonization in the liver and spleen. Collectively, these findings demonstrate that LiaS acts as a pivotal regulatory factor, orchestrating both environmental stress adaptation and virulence traits in *L. monocytogenes*.

In conclusion, LiaS possesses multiple functions in stress response resistance and pathogenicity and has a significantly positive effect on pH stress, osmotic pressure, oxidative stress, heavy metal, motility, and pathogenicity (Figure 6). This study provides valuable insights into the mechanisms of the LiaS two-component system in the stress response and pathogenicity of *L. monocytogenes* and benefits microbial pathogenesis, infection biology, and the interaction between host and pathogen.

## Supplementary Material

Figure_supplementary.docx

Author Checklist Full.pdf

KVIR]_OpenScienceForm.docx

## Data Availability

The images directly generated by taking photos or software are visible in the main text or the supplementary file. And the original data for drawing line charts, bar charts, etc., are visible in the Figshare link. The dataset that supports the findings of this study is available on the figshare database under https://doi.org/10.6084/m9.figshare.29243033.

## Open scholarship

This article has earned the Center for Open Science badge for Open Data. The data are openly accessible at https://doi.org/10.1080/00224545.2023.2195992
