# Supplementary material for: The two-component system histidine kinase LiaS contributes to the stress resistance and virulence of zoonotic Listeria monocytogenes
Source: Virulence. 2026 Jul 21;17(1):2707724. doi: 10.1080/21505594.2026.2707724 (PMC13432813; doi:10.1080/21505594.2026.2707724)
Supplement: Figure_supplementary.docx [file KVIR_A_2707724_SM3159.docx]

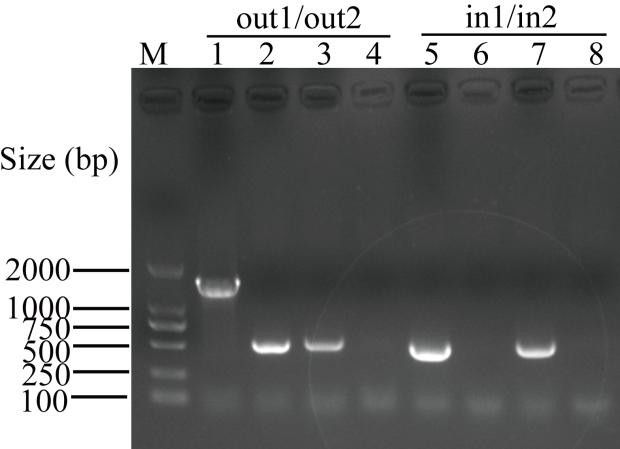


Figure S1. Verification of the ∆*liaS*, C∆*liaS* by PCR. Lane 1 and 5 are the are the PCR verification of EGD-e, lane 2 and 6 are the are the PCR verification of ∆*liaS* strains, lane 3 and 7 are the PCR verification of C*∆liaS* strains, lane 4 and 8 are negative control. M: DL 2000 DNA marker.


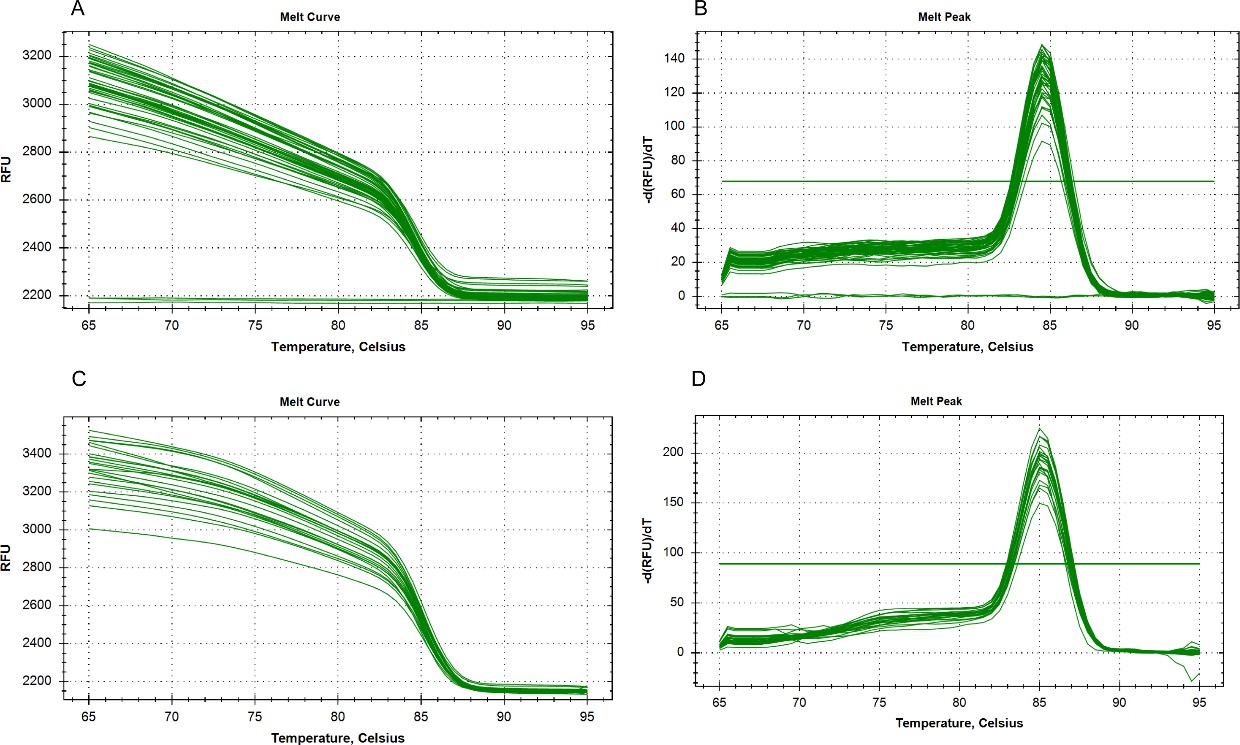


Figure S2. Amplification curve images of qPCR for osmotic stress-related genes. Amplification curve images of qPCR in pH7.0 condition (A-B); Amplification curve images of qPCR in pH 3.0 condition (C-D).
